# Supplementary material for: Phase separation properties of RPA combine high-affinity ssDNA binding with dynamic condensate functions at telomeres
Source: Nat Struct Mol Biol. 2023 Mar 9;30(4):451–62. doi: 10.1038/s41594-023-00932-w (PMC10113159; doi:10.1038/s41594-023-00932-w)
Supplement: Supplementary file 1 — Supplementary Figures 1–4, legends to Supplementary Videos 1 and 2 and Supplementary Tables 1–6, Methods, References, list of Source Data files and Uncropped Blot Source Data for Supplementary Figure 2. [file 41594_2023_932_MOESM1_ESM.pdf]

nature structural & molecular biology

Article https://doi.org/10.1038/s41594-023-00932-w

Phase separation properties of RPA combine high-affinity ssDNA binding with dynamic condensate functions at telomeres

In the format provided by the authors and unedited

Supplementary Information

Phase separation properties of RPA combine high-affinity ssDNA binding with dynamic condensate functions at telomeres

Vincent Spegg[1], Andreas Panagopoulos[1], Merula Stout[1], Aswini Krishnan[1], Giordano Reginato[2,3], Ralph Imhof[1], Bernd Roschitzki[4], Petr Cejka[2,3], Matthias Altmeyer[1,*]

[1] Department of Molecular Mechanisms of Disease, University of Zurich (UZH), Zurich, Switzerland
[2] Institute for Research in Biomedicine, Faculty of Biomedical Sciences, Università della Svizzera italiana (USI), Bellinzona, Switzerland
[3] Department of Biology, Institute of Biochemistry, Eidgenössische Technische Hochschule (ETH), Zurich, Switzerland
[4] Functional Genomics Center Zurich, University of Zurich (UZH) & Eidgenössische Technische Hochschule (ETH), Zurich, Switzerland

* Correspondence: matthias.altmeyer@uzh.ch

Inventory

Supplementary Figures S1 to S4 with Figure Legends
Legends to Movies S1 and S2
Legends to Tables S1 to S6
Supplementary Methods
References to Supplementary Information
List of Source Data Files
Uncropped Blot Source Data Supplementary Figure 2

Supplementary Figures

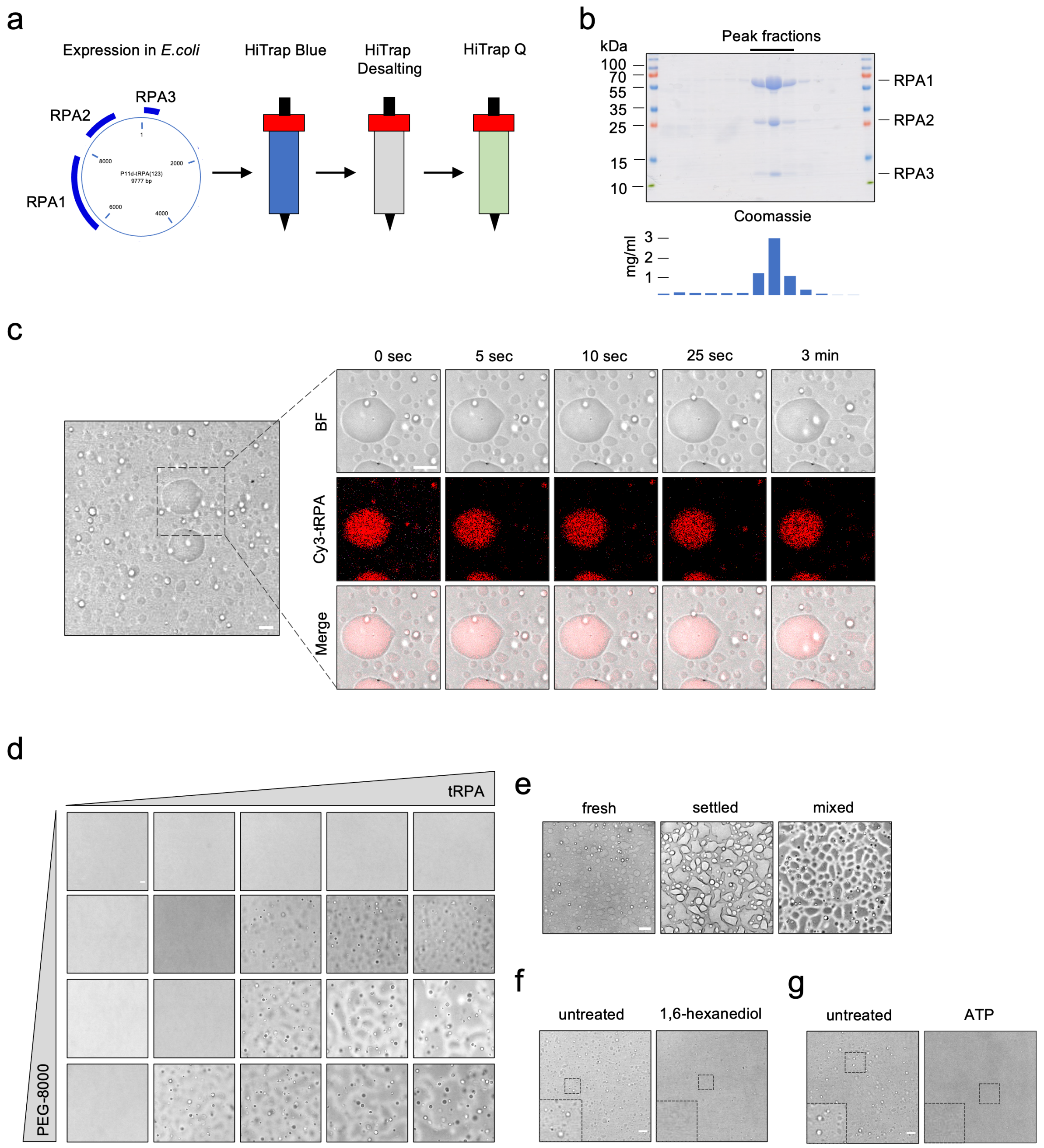

Supplementary Figure S1. Purified heterotrimeric RPA forms liquid droplets *in vitro*. (a) The three RPA subunits were expressed in *E. coli* and purified over three sequential chromatography columns as indicated. **(b)** Coomassie staining of the final RPA fractions collected after elution from the HiTrap Q column. Relative protein yields are indicated below. **(c)** Cy3-labeled purified RPA (labeled:unlabeled ratio 1:9) spontaneously forms liquid droplets *in vitro*. Representative stills from time-lapse microscopy are shown. **(d)** RPA phase separation and surface wetting in conditions of increasing RPA and PEG-8000 concentrations (RPA: 0 µM, 5 µM, 7.5 µM, 10 µM, 12.5µM; PEG-8000: 0 %, 7.5 %, 10 %, 12.5 %.). **(e)** RPA droplet formation, surface wetting and remixing. RPA droplets were allowed to settle for 30 min prior to remixing. **(f)** RPA droplet sensitivity to 3% 1,6-hexanediol. **(g)** RPA droplet sensitivity to 4 mM ATP. Scale bars 10 µm.

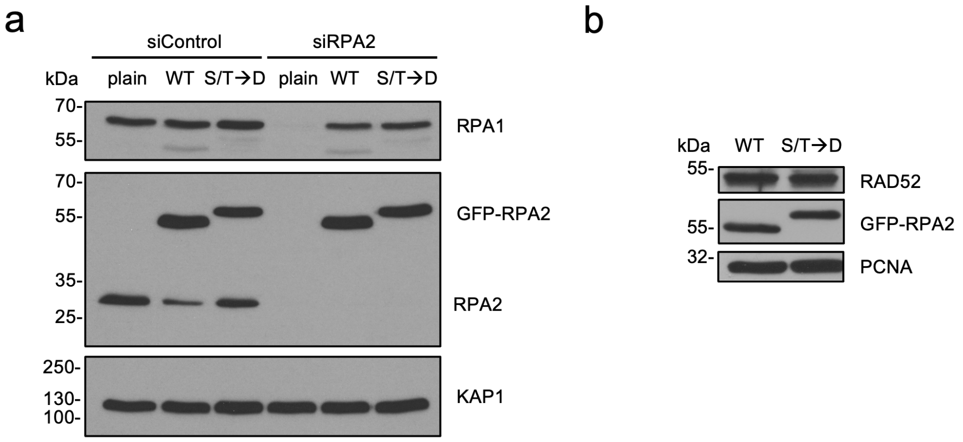

Supplementary Figure S2. RPA and RAD52 expression in siRNA-resistant GFP-RPA2 cell lines. **(a)** Western blot analysis of U-2 OS cells stably expressing siRNA-resistant GFP-RPA2 WT or S/T→D. Cells were treated for 72h with siRNA as indicated. Endogenous RPA2 and GFP-RPA2 were detected with an anti-RPA2 antibody. **(b)** RAD52 levels are comparable in U-2 OS cells expressing RPA2 WT or S/T→D after depletion of endogenous RPA2 for 72h.

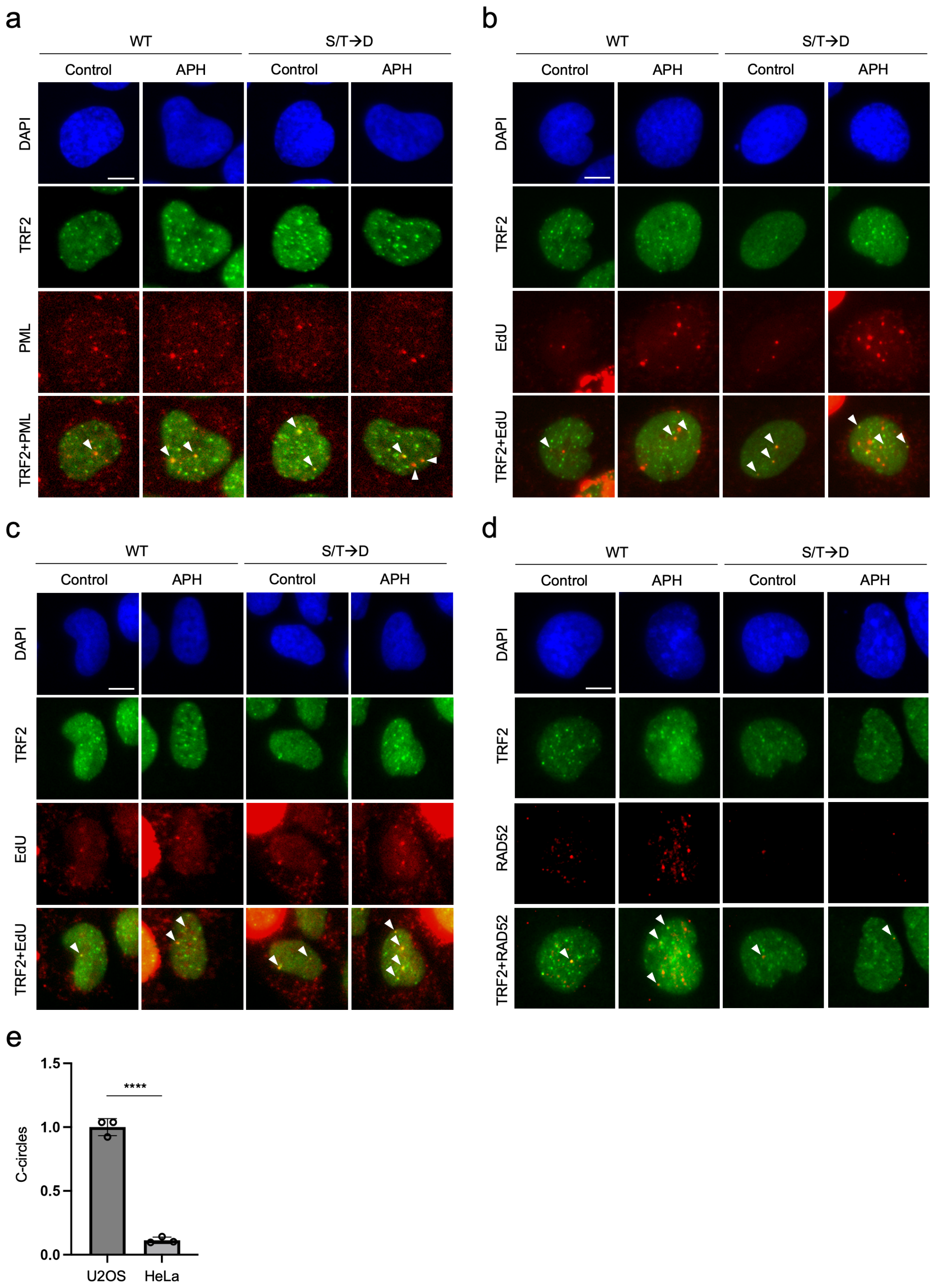

Supplementary Figure S3. APBs and telomere synthesis outside S-phase. (a) Representative fluorescence microscopy images of U-2 OS cells stably expressing siRNA-resistant GFP-RPA2 WT or S/T→D, depleted for endogenous RPA2, and treated as indicated. Cells were stained for TRF2 and PML and APBs were scored. **(b)** Representative fluorescence microscopy images of U-2 OS cells stably expressing siRNA-resistant GFP-RPA2 WT or S/T→D, depleted for endogenous RPA2, and treated as indicated. Cells were stained for TRF2 and EdU. G2 cells were identified by QIBC based on DAPI and

EdU, and EdU incorporation at TRF2 foci was scored. **(c)** Representative fluorescence microscopy images of U-2 OS cells stably expressing siRNA-resistant GFP-RPA2 WT or S/T→D, depleted for endogenous RPA2, and treated as indicated. Cells were stained for TRF2 and EdU. G1 cells were identified by QIBC based on DAPI and EdU, and EdU incorporation at TRF2 foci was scored. **(d)** Representative fluorescence microscopy images of U-2 OS cells stably expressing siRNA-resistant GFP-RPA2 WT or S/T→D, depleted for endogenous RPA2, and treated as indicated. Cells were stained for TRF2 and RAD52. RAD52 foci and their co-localization with TRF2 was scored. **(e)** Relative C-circle levels in ALT-positive U-2 OS cells compared to ALT-negative HeLa cells as control for the C-circle assay. C-circle levels were determined by qPCR and normalized to the average C-circle level of U-2 OS cells. Average and standard deviations from n=3 replicates are shown. Two-tailed unpaired t-test, **** $p<0.0001$. Scale bars 10 μm.

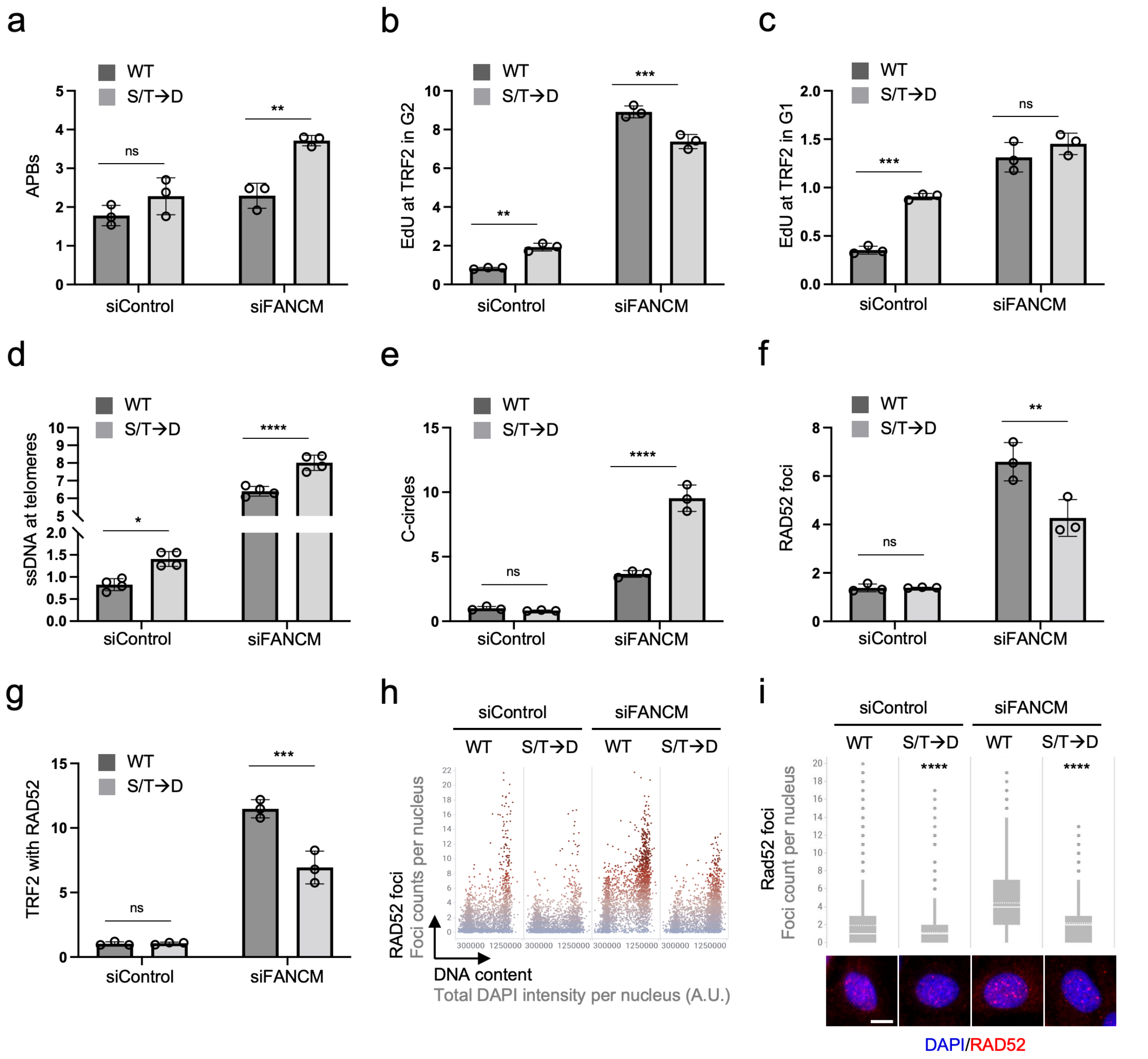

Supplementary Figure S4. RPA condensation is linked to telomere maintenance. (a) U-2 OS cells stably expressing siRNA-resistant GFP-RPA2 WT or S/T→D, depleted for endogenous RPA2 and treated with siRNA against FANCM as indicated were stained for PML and TRF2 to quantify APBs. Averages and standard deviation from n=3 independent samples with 50 cells per replicate are shown. Two-way ANOVA with Šidák`s test, siControl ns p=0.1815, siFANCM ** p=0.0013. **(b)** Cells treated as in (a) were EdU labeled, stained for TRF2 and gated according to their cell cycle position based on their total DAPI and mean EdU intensities. G2 cells were analyzed for EdU-positive TRF2 foci. Averages and standard deviation from n=3 independent samples are shown for 100 G2 cells per replicate. Two-way ANOVA with Šidák`s test, siControl ** p=0.0018, siFANCM *** p=0.0002. **(c)** As in (b) for G1 cells. Averages and standard deviation from n=3 independent samples are shown for 100 G1 cells per replicate. Two-way ANOVA with Šidák`s test, siControl *** p=0.0002, siFANCM ns p=0.2278. **(d)** Cells were treated as in (a), and non-denaturing TelG-FISH was performed to detect telomeric ssDNA. Averages and standard deviation from n=4 independent samples are shown (cell number: $WT_{siControl}$ n_1=634, n_2=586, n_3=589, n_4=617; $WT_{siFANCM}$ n_1=585, n_2=578, n_3=590, n_4=617; $S/T{\rightarrow}D_{siControl}$ n_1=639, n_2=593, n_3=613, n_4=604; $S/T{\rightarrow}D_{siFANCM}$ n_1=585, n_2=575, n_3=603, n_4=581). Two-way ANOVA with Šidák`s test, siControl * p=0.0244, siFANCM **** p<0.0001. **(e)** Cells were treated as in (a) and C-circles were determined by qPCR, normalized to RPA2 WT control. Average and standard deviations from n=3 replicates are shown. Two-way ANOVA with Šidák`s test, siControl ns

p=0.9102, siFANCM **** p<0.0001. **(f)** Cells were treated as in (a) and RAD52 foci counts were manually quantified. Two-way ANOVA with Šidák`s test, siControl ns p=0.9998, siFANCM ** p=0.0018. **(g)** Cells were treated as in (a) and RAD52-positive TRF2 foci were scored. Two-way ANOVA with Šidák`s test, siControl ns p=0.9982, siFANCM *** p=0.0001. **(f-g)** Averages and standard deviations from n=3 independent samples are shown (cell number: $WT_{siControl}$ n_1=134, n_2=145, n_3=1115; $WT_{siFANCM}$ n_1=80, n_2=68, n_3=72; S/T→$D_{siControl}$ n_1=117, n_2=108, n_3=114; S/T→$D_{siFANCM}$ n_1=78, n_2=83, n_3=65). **(h)** Example of cell cycle resolved RAD52 foci formation in response to ALT activation by FANCM depletion in U-2 OS cells expressing RPA WT or S/T→D. RAD52 foci counts were analyzed by QIBC (cell number: $WT_{siControl}$ n=2708; $WT_{siFANCM}$ n=2678; S/T→$D_{siControl}$ n=2794; S/T→$D_{siFANCM}$ n=2763). **(i)** Quantification of RAD52 foci counts from (h) (cell number: $WT_{siControl}$ n=2708; $WT_{siFANCM}$ n=2678; S/T→$D_{siControl}$ n=2794; S/T→$D_{siFANCM}$ n=2763). Box plots with medians (solid horizontal lines) and means (dashed horizontal lines) are shown. Box limits indicate the 25th percentile (Q1) and 75th percentile (Q3) and box represents the interquartile range (IQR, Q3-Q1). Whiskers define the lower and upper adjacent value. Dots represent outliers that are greater than Q3+1.5xIQR. One-way ANOVA with Tukey test, siControl **** p<0.0001, siFANCM **** p<0.0001. Representative images are shown below. Scale bars 10μm.

Legends to Movies S1 and S2

Movie S1. Cry2-mCherry-RPA2 optoDroplet formation. Cry2-mCherry-RPA2 was transiently expressed in U-2 OS cells for 24 hours and clustering was induced by blue light. Cells were imaged at 15 seconds intervals for 6 minutes. A representative cell, corresponding to Figure 1d, is shown. Scale bar 10μm.

Movie S2. Trimeric RPA droplet formation. Trimeric RPA was purified, and RPA droplet formation was analyzed *in vitro* by time-lapse microscopy at 5 seconds intervals for 20 minutes. A representative field of view, corresponding to Figure 2b, is shown. Scale bar 10μm.

Legends to Supplementary Tables S1 and S6

Table S1. TurboID-RPA2 mass spectrometry. Proteins significantly enriched or depleted in TurboID-RPA2 light vs. TurboID-RPA2 dark conditions (p-value ≤ 0.05, fold-change ≥ 1.5). Related to Figure 5. p-values were assessed by moderated t-test.

Table S2. TurboID-RPA2 mass spectrometry. List of all proteins identified by TurboID proximity labeling mass spectrometry.

Table S3. Oligonucleotides. Oligonucleotides used for *in vitro* RPA droplet formation and turbidity experiments together with their sequences.

Table S4. Cell lines. List of all cell line sources used in this study.

Table S5. Primers. List of cloning and qPCR primers used in this study.

Table S6. Plasmids. List of plasmids used in this study.

Supplementary Methods

Cloning of Cry2-mCherry-fusion constructs

The Cry2-mCherry construct[1] was linearized with primers 1 and 2 and used as a backbone for Gibson assembly to generate fusion proteins. The inserts were generated using the following primers: GST, primers 3 and 4; FUS_N, primers 5 and 6; RPA1, primers 7 and 8; RPA2, primers 9 and 10; RPA3, primers 11 and 12; RNF8, primers 13 and 14; RNF168, primers 15 and 16; CtIP, primers 17 and 18; RAD51, primers 19 and 20. The polycistronic Cry2-mCherry-RPA2-P2A-RPA3-T2A-RPA1 plasmid was cloned stepwise using Gibson assembly by first generating an Cry2-mCherry-RPA2-P2A-RPA3 vector (primers 1, 21, 22, 23) followed by adding T2A-RPA1 (primers 1, 24, 25, 26) using the single Cry2-mCherry-RPA constructs as a template.

An SV40-NLS signal was added to Cry2-mCherry-RPA2 by site-directed mutagenesis (primers 27 and 28) to ensure nuclear localization. This construct was then used to linearize Cry2-mCherry-NLS (primers 29 and 2), which served as a backbone for Gibson assembly to generate RPA2 S/T→D+NLS (primers 30 and 31, using p11d-tRPA-32Asp8 (Addgene plasmid #102617) as template), RPA2 S→A+NLS (primers 32 and 31, using p11d-tRPA-32Ala9 (Addgene plasmid #102616) as template) , and yeast Rfa2+NLS (primers 33 and 34, using p11d-sctRPA (Addgene plasmid #102614) as template). The RPA2 phosphomutants were generated by site-directed mutagenesis using RPA2+NLS and the following primers: RPA2 S4D, primers 35 and 36; RPA2 S8D, primers 37 and 38; RPA2 S11D, primers 39 and 40; RPA2 S12D, primers 41 and 42; RPA2 T21D, primers 43 and 44; RPA2 S23D, primers 45 and 46; RPA2 S33D, primers 47 and 48; RPA2 S11/12/13D, primers 49 and 50. RPA2 S23/33D was generated using RPA2 S33D and primers 51 and 52. RPA2 S8/33D was generated using RPA2 S8D and primers 47 and 48. RPA2 S4/8D was generated using RPA2 S8D and primers 53 and 54. RPA2 T21D/S33D was generated using RPA2 T21D and primers 47 and 48. RPA2 S8D/T21D/S33D was generated using RPA2 T21D/S33D and primers 37 and 38. RPA2 T21D/S29/33D was generated using RPA2 T21D/S33D and primers 55 and 56. RPA2 T21D/S23/29/33D was generated using RPA2 T21D/S29/33D and primers 57 and 58.

Cloning of TurboID-RPA2-mCherry-Cry2

The TurboID-mCherry-Cry2 plasmid was kindly provided by Angelos Constantinou[2,3]. The plasmid was linearized using primers 59 and 60 and RPA2 was amplified from Cry2-mCherry-RPA2 using primers 61 and 62 and inserted by Gibson assembly.

Cloning of GFP-RPA2 mutants

The pAC-GFP-C1 plasmid (Takarabio, 632470) was linearized with primers 63 and 64. RPA2 wildtype was inserted from Cry2-mCherry-RPA2 with primers 65 and 66, RPA2 S/T→D was inserted from Cry2-mCherry-RPA2 S/T→D with primers 66 and 67 by Gibson assembly. The resulting GFP-RPA2 plasmids were rendered siRNA-resistant by site-directed mutagenesis using primers 68 and 69.

Cloning of TRF2-RFP

The pCIBN-hTRF2-tagRFP-T plasmid (Addgene plasmid #103812, kindly provided by Karsten Rippe[4]) was linearized with primers 70 and 71. A hygromycin resistance cassette was amplified from pTRE2hyg (Takarabio, 631014) with primers 72 and 73 and inserted via Gibson assembly. The N-terminal CIB1 was removed by linearization of the resulting plasmid using primers 74 and 75 followed by blunt end re-ligation.

Cloning of p11d-tRPA-32ΔN

To express tRPA lacking the disordered N-terminal domain of RPA2, the sequence coding for amino acids 1-42 of RPA2 were removed by linearization of p11d-tRPA(123) using primers 76 and 77 followed by blunt end re-ligation.

Generation of stable cell lines

For the generation of U-2 OS GFP-RPA2 cell lines, U-2 OS cells were transfected with the desired plasmids using FuGENE HD transfection reagent (Promega) according to the manufacturer's

instructions. 48 h after transfection, the media was exchanged for selection media (DMEM containing 10% fetal bovine serum (Gibco), 1% penicillin-streptomycin antibiotics, and 400 µg/ml Geneticin). After selection, the cell lines were sorted on a FACS Aria III 3L system equipped with a 488 nm laser line for homogenous GFP-RPA2 expression. GFP-RPA2 expression levels and siRNA-resistance were validated by Western blot and QIBC. For the generation of the U-2 OS GFP-RPA2 / TRF2-RFP cell line, U-2 OS GFP-RPA2 cells were transfected with the desired plasmid using FuGENE HD transfection reagent (Promega) according to the manufacturer's instructions. 48 h after transfection, the media was exchanged for selection media (DMEM containing 10% fetal bovine serum (Gibco), 1% penicillin-streptomycin antibiotics, 100 µg/ml Hygromycine B (ThermoFisher Scientific) and 400 µg/ml Geneticin). After selection, the cell line was sorted on a FACS Aria III 3L system equipped with a 488 nm and 561 nm laser lines for double positive GFP-RPA2 and TRF2-RFP expression. GFP-RPA2 and TRF2-RFP expression levels and functionality were validated by immunofluorescence staining and QIBC analysis. For the generation of U-2 OS TurboID cell lines, U-2 OS Flp-In T-REx cells were transfected with desired plasmids using jetPRIME transfection reagent (Polyplus-transfection) according to the manufacturer's instructions. 48 h after transfection, the media was exchanged for selection media (DMEM containing 10% fetal bovine serum (Gibco), 1% penicillin-streptomycin antibiotics, and 100µg/ml Hygromycine B (ThermoFisher Scientific)). After clonal selection and amplification, correct integration of the plasmids was confirmed by blue light-induced optoDroplet formation and by Western blot analysis of biotin-labeling in nuclear and whole cell extracts.

Fluorescence recovery after photobleaching (FRAP)
For FRAP experiments in cells, U-2 OS GFP-RPA cells were seeded into a 96 well imaging plate (Greiner µclear). GFP-RPA foci were analyzed as indicated under conditions of endogenous replication stress, after treatment with 1µM ATR inhibitor AZ-20 (Tocris) for 24h, and after 4h recovery from 4 Gy ionizing irradiation (IR) generated by a Faxitron Cabinet X-ray System Model RX-650. FRAP analyses were performed at a Leica SP5 UV-VIS inverted confocal laser scanning microscope (Leika Application Suite X 3.5.7.23225) using a 63x HCX PL APO (NA 1.4) oil objective and a 488nm Argon laser. Five pre-bleach images were acquired before bleaching regions of interest (i.e. individual GFP-RPA foci) for five consecutive cycles at 100% laser power. 60 images post bleaching were acquired at 0.392s intervals and FRAP of GFP-RPA foci was analyzed in 10-18 cells per condition.
For FRAP experiments *in vitro*, freshly purified trimeric RPA WT or S/T→D was incubated with 40 nucleotide FAM-labelled ssDNA at a final concentration of 7.5 µM at room temperature in diluted Sørensen buffer (43.4 mM Na_2HPO_4, 6.6 mM KH_2PO_4, pH 7.6) supplemented with 150 mM KCl, 25 µM EDTA, and 1 mM DTT in 4% PEG-8000. FRAP analyses were performed at a Leika SP8 inverse FALCON confocal laser scanning microscope (Leika Application Suite X 3.5.7.23225) using a 63x HC PL APO corr CS2 oil objective (NA 1.4) and a 488 nm white light laser. Five pre-bleach images were acquired before bleaching regions of interest for five consecutive cycles with 100 % laser power of the 488 nm and 471 nm white light laser and 100 % laser power of the 405 nm diode laser. 40 images post bleaching were acquired at 10s intervals and FRAP was analyzed in 15 droplets or aggregates per condition.

RPA foci sensitivity to 1,6-hexanediol
For live cell imaging, U-2 OS GFP-RPA cells were seeded into a 96 well imaging plate (Greiner µclear) and treated with 0.2 µM Aphidicolin (Sigma-Aldrich) for 24 h. Time-lapse microscopy was performed in temperature and CO_2 controlled conditions (37 °C, 5% CO_2) on a GE Healthcare IN Cell Analyzer 2500HS using a CFI Plan Apo Lambda 20x (NA 0.75) air objective. Prior to 2.5% 1,6-hexanediol (Sigma-Aldrich) treatment, cells were imaged at 20s intervals for 2 min. After 1,6-hexanediol addition, cells were followed at 20s intervals for 5 min. For QIBC analysis, U-2 OS GFP-RPA cells were grown on sterile 12 mm glass coverslips and treated for 24 h with 0.2 µM Aphidicolin. Cells were then treated with 1.25%, 2.5% or 5% 1,6-hexanediol for 5 min prior fixation in 3% formaldehyde in PBS for 15 min at room temperature. Cells were washed once in PBS, permeabilized for 5 min at room temperature in 0.2% Triton X-100 (Sigma-Aldrich) in PBS and washed twice in PBS. Coverslips were then incubated for 10 min with PBS containing 4',6-Diamidino-2-Phenylindole Dihydrochloride (DAPI, 0.5 µg/ml) at room temperature. Cells were subsequently washed three times in PBS and briefly submerged in

distilled water prior to being mounted on glass slides with 6µl Mowiol-based mounting media (Mowiol 4.88 in Glyerol/TRIS). Imaging and QIBC analysis were performed as described above.

Quantification of APBs
For APB quantification, U-2 OS GFP-RPA2 WT and S/T→D cells were stained for PML and TRF2 and images were acquired at the Olympus ScanR Screening System (ScanR Acquisition 3.01) with a 20x UPLSAPO (NA 0.75) air objective as described in the QIBC section. At least 50 cells per replicate were quantified in triplicates for the analysis of PML-positive TRF2 foci.

Quantification of DNA synthesis at telomeres
For detection of DNA synthesis at telomeres in G1 and G2 cells, samples were pulsed with 100 µM EdU for 1 h prior fixation, followed by detection of EdU and an immunofluorescence staining against TRF2. Images were acquired at the Olympus ScanR Screening System using a 20x UPLSAPO (NA 0.75) air objective as described in QIBC section. To determine cell cycle phases, cell cycle gating was performed using nuclear total DAPI intensity and mean intensity of the EdU signal as shown in Extended Data Figure 9a. TRF2 foci overlapping with EdU foci were quantified in triplicates from 100 G1 or G2 cells per replicate.

Quantification of RPA at telomeres
For the localization of RPA at telomeres, U-2 OS GFP-RPA2 WT and S/T→D cells were stained for TRF2, and images were acquired at the Olympus ScanR Screening System with a 20x UPLSAPO (NA 0.75) air objective as described in the QIBC section. At least 50 RPA2/TRF2 foci positive cells per replicate were quantified in triplicates for the analysis of co-localization between both markers.

Quantification of RAD52 at telomeres
For the localization of RAD52 at telomeres, U-2 OS GFP-RPA2 WT and S/T→D cells were stained for RAD52 and TRF2. Imaging was performed at the Olympus ScanR Screening System using a 20x UPLSAPO (NA 0.75) air objective as described in the QIBC section. At least 65 RAD52/TRF2 foci positive cells per replicate were quantified in triplicates for the analysis of co-localization between RAD52 and TRF2.

Image analysis with Fiji
Manual image analysis and quantification was performed using Fiji/ImageJ 64-bit, Version 2.00-rc54/1.51h

Detection of ssDNA at telomeres by native FISH
Native FISH was performed as described previously[5]. Specifically, cells were seeded and treated in 96 well plates (Ibidi) and then fixed in 4% formaldehyde in PBS for 10 min at room temperature, washed twice in PBS, permeabilized for 5 min at room temperature in 0.2% Triton X-100 (Sigma-Aldrich) in PBS, and washed three times in PBS. Samples were dehydrated in 70%, 95% and 100% ethanol for 5 min each at room temperature, and then air-dried. 90 µl of hybridization mix (10 mM Tris-HCl pH 7.4, 70% formamide, 0.5% blocking reagent (Roche, 11096176001), TelG-Cy5 probe ($(TTAGGG)_n$, PNA Bio, F1007, 1:2000)) were added, followed by hybridization for 3 h at room temperature. Samples were then washed twice with wash buffer A (10 mM Tris-HCl pH 7.4, 70% formamide) for 15 min, and three times with wash buffer B (100 mM Tris-HCl pH 7.4, 150 mM NaCl, 0.08% Tween-20) for 5 min. Next, samples were incubated for 10 min with PBS containing DAPI (0.5 µg/ml), washed three times with PBS, and then stored in PBS. QIBC was performed on an Olympus IXplore SpinSR10 real time spinning disk confocal imaging system equipped with a Yokogawa CSU-W1 confocal scanner unit, a motorized stage, Olympus real-time controller (U-RTCE) and Z-drift compensator (IX3-ZDC2), laser lines at 405 nm (50 mW), 488 nm (100 mW), and 640 nm (100 mW), and a 2x Hamamatsu ORCA-Fusion sCMOS camera system (2304 x 2304 pixels, pixel size 6.5 x 6.5 µm). A 40x UPLAN S Apo (NA 0.95) air objective was used. Multiple positions with 30 z-layers were acquired using Olympus ScanR Image Acquisition Software (version 3.2). For image analysis using the ScanR analysis software 3.2, a dynamic background correction was applied, and nuclei segmentation was performed using an integrated intensity-based object detection module based on the DAPI signal. Foci segmentation was

performed using an integrated spot-detection module. Downstream analyses were focused on properly detected interphase nuclei containing a 2N-4N DNA content as measured by total and mean DAPI intensities, and comparable GFP expression levels. Within each experiment, comparable cell numbers per condition were analyzed.

Stimulated emission depletion (STED) microscopy

U-2 OS cells stably expressing GFP-RPA2, depleted for endogenous RPA2 for 72h and treated for 24h with 0.2 μM Aphidicolin, were used for STED microscopy at a Leica DMI6000B SP8 inverse STED 3x confocal system (Leika Application Suite X3.5.7.23225) equipped with a 93x HC PL APO STED WHITE motCORR glycerol objective (NA 1.3). GFP was excited with a 488nm continuous white light laser and STED was performed using a continuous 592nm laser with a gating time of 0.7-6 ns. Images (1568x1568 pixels) with a 20 nm pixel size were acquired with the pinhole set to 1.0 A.U. and a scan rate of 400 Hz and a dwell time of 375 ns. For image acquisition a HyD detector (495-550 nm) was used in photon-counting mode, and eight line accumulations were applied.

C-circle analysis

GFP-RPA2 WT and S/T→D cells were depleted of endogenous RPA2 alone or in combination with FANCM by siRNA for 72h. Samples were harvested by trypsinization and the pellets snap-frozen in liquid nitrogen prior to storage at -20°C. Genomic DNA extraction and C-circle amplification were performed as previously described[6]. In short, genomic DNA was extracted from cell pellets using the QIAmp DNA FFPE Tissue Kit (Qiagen) and the DNA concentration was measured using a NanoDrop ND-1000 Spectrophotometer. For C-circle amplification, 30 ng of gDNA as input with or without phi29 polymerase (ThermoFisher Scientific) were used. C-circle levels were measured by quantitative PCR (qPCR)[7] by quantification of single stranded repetitive telomeric sequences (5'-TTAGGG-3') in triplicates with KAPA SYBR FAST qPCR Kit (KAPA Biosystems) on a Rotor-Gene Q system (Qiagen, Rotor-Gene Q Series Software 20.4.21) using primers qPCR 1-4 (Table S5). C-circle levels were normalized with the 36B4 single copy gene product. For relative C-circle quantification, the delta-delta Ct was calculated between the phi29 amplified and non-amplified samples.

RPA fusion analysis by live cell microscopy

U-2 OS cells expressing endogenously tagged mScarlet-RPA1 and U-2 OS GFP-RPA2 cell lines depleted of endogenous RPA2 were seeded into a 96 well imaging plate (Ibidi). 24h after seeding, the medium was exchanged to FluoroBrite DMEM Glutamax supplemented with 10% fetal bovine serum (Gibco) and Glutamax (ThermoFisher Scientific) and, where indicated, cells were treated with 0.2 μM Aphidicolin (Sigma-Aldrich) and inhibitors against ATM (10 μM KU55933, Tocris), ATR (1 μM AZ-20, MedChemExpress), DNA-PK (10 μM Nu7441, Tocris), and CDK1/2 (10 μM AZD5438, Tocris). Time-lapse microscopy was carried out in temperature and CO_2 controlled conditions (37 °C, 5% CO_2) on the GE Healthcare IN Cell Analyzer 2500HS (V7.4) using a CFI Plan Apo Lambda (NA 0.75) air objective at 20 min intervals for 48 h. Foci fusion and fission events were scored manually from 100 cells per condition for each replicate.

GFP-RPA2 expression levels were quantified using the Olympus ScanR Image Analysis Software (version 3.0.1), a dynamic background correction was applied, and nuclei segmentation was performed using an integrated intensity-based object detection module based on the nuclear GFP signal.

Metaphase FISH analysis

Metaphase FISH was performed as described previously[5]. U-2 OS GFP-RPA2 WT and S/T→D cells were depleted of endogenous RPA2 by siRNA for 72h. Cells were treated for 3h with 0.1 μg/ml KaryoMAX Colcemid (ThermoFisher Scientific) prior to cell harvesting by trypsinization. Cells were washed once with PBS and pelleted by centrifugation at 200 x g for 5 min. Cell pellets were resuspended in 5 ml pre-warmed hypotonic solution (75 mM KCl) followed by a 20 min incubation in standard cell culture conditions (37 °C, 5% CO_2, humidified atmosphere). After addition of 1.25 ml of fixative solution (75% methanol, 25% acetic acid), cells were incubated 10 min at 37 °C, pelleted by centrifugation at 200 x g for 5 min and the supernatant was removed. After two rounds of fixation, cells were resuspended in fixative solution and spread on glass slides (Thermo Scientific). After incubation of the spreads overnight at room temperature, samples were dehydrated by incubation in 70%, 95% and

100% ethanol for 5 min each at room temperature, and then air-dried. Onto each glass slide 30µl of hybridization mix (10 mM Tris-HCl pH 7.4, 70% formamide, 0.5% blocking reagent (Roche, 11096176001), TelC-Cy3 probe ($(CCCTAA)_n$, PNA Bio, F1002, 1:1000)) was added, followed by denaturing at 80°C for 10 min and hybridization for 3h at room temperature. Samples were washed twice with wash buffer A (10 mM Tris-HCL pH 7.4, 70% formamide) for 15 min, and three times with wash buffer B (100 mM Tris-HCl pH 7.4, 150mM NaCl, 0.08% Tween-20) for 5 min. The second wash contained DAPI (0.5 µg/ml). Finally, samples were dehydrated by incubation in 70%, 95% and 100% ethanol for 5 min each at room temperature, before mounting using 20 µl ProLong Gold Antifade (Invitrogen). Images were acquired using a 63x HC PL APO CS2 oil objective (NA 1.4) on a Leika Thunder Imager 3D Live Cell widefield microscope (Leika Application Suite X 3.7.5.24914) equipped with a Leica LED8 light source for illumination at 390 nm and 555 nm, a DFT5 Quad filter, and a Leica monochrome fluorescence DFC9000 GTC camera system (2048x2048 pixels, pixel size 6.5x6.5 µm). Telomere loss was quantified manually from biological triplicates with a total of 32-39 metaphases and 1823-2007 scored chromosomes per condition.

***In vitro* phosphorylation**

Purified trimeric RPA was phosphorylated *in vitro* using the DNA-PK Kinase Enzyme System (Promega) in the presence of 0.5 mM ATP according to the manufacturer's instructions. Successful RPA phosphorylation was verified by Western blot using a phosphospecific antibody against RPA2 pS4/8 (rabbit, Bethyl, A300-245, 1:500).

TurboID sample preparation for proximity labeling mass spectrometry

U-2 OS TurboID-mCherry-Cry2 and U-2 OS TurboID-RPA2-mCherry-Cry2 cells were seeded into 15cm cell culture dishes. 24h prior to blue light exposure, cells were incubated with doxycycline (1µg/ml) to induce construct expression. Cells were either kept in the dark as negative control condition or exposed to 30 cycles of 5s blue light followed by 25s without light in a custom-made blue light equipped with 8 x 1W LED lamps with a power of 500 Lm in 10 cm distance to the cells. Biotin labeling was performed for 15 minutes (i.e. together with the light activation) with 500 µM biotin (Sigma-Aldrich). Cells were harvested by trypsinization, and cell pellets were resuspended in a buffer containing 10 mM EDTA, 10 mM KCl, 10 mM Hepes, 1x protease inhibitor cocktail (Roche), 1 mM DTT, and 0.1% NP-40, followed by an incubation for 10 min on ice before centrifugation at 10000 x g for 10 min at 4 °C. The nuclear pellet was resuspended in a buffer containing 1 mM EDTA, 0.4 M NaCl, 0.02 M Hepes, 1% Glycerol, 1 mM DTT, 1x protease inhibitor cocktail and incubated on a Thermomixer at 4 °C for 2h. Nuclear extracts were centrifuged for 10 min at 21100 x g and the supernatants used for further steps.

After protein concentrations were measured by Bradford, nuclear extracts were incubated with Dynabeads-M280 Streptavidine beads (ThermoFisher Scientific) overnight at 4 °C in a binding buffer containing 20 mM Hepes, 20% Glycerol, 2 mM $MgCl_2$, 0.2 mM EDTA, 0.5% NP-40, 150 mM NaCl, 1x protease inhibitor cocktail, and 0.5 mM DTT. Beads were then washed 2x in binding buffer, 2x in PBS containing 0.5% NP-40, and 2x in PBS only. For each sample, the washed beads were re-suspended in 45 µl digestion buffer (10 mM Tris/HCl, 2 mM $CaCl_2$, pH 8.2) and the proteins were digested using 5 µl of sequencing grade trypsin (100 ng/µl in 10 mM HCl, Promega). The digestion was carried out in a microwave instrument (Discover System, CEM) for 30min at 5W and 60 °C. The supernatant was collected in a fresh tube and the magnetic beads were washed with 150 µl of 0.1% TFA / 50% acetonitrile and combined with the previously collected supernatant. The samples were finally dried in a speed-vac, resolubilized in 20 µl of 3% acetonitrile, 0.1% formic acid spiked with iRT peptides (Biognosys). Six replicates per condition were analyzed by liquid chromatography mass spectrometry.

Liquid chromatography mass spectrometry analysis

Mass spectrometry analysis was performed on a Q Exactive mass spectrometer (Thermo Scientific) equipped with a Digital PicoView source (New Objective) and coupled to a nanoAcquity UPLC (Waters). Solvent composition at the two channels was 0.1% formic acid for channel A and 0.1% formic acid in acetonitrile for channel B. For each sample 1µl of peptides were loaded on a trap column Symmetry C18 (100Å, 5µm, 180µm x 20mm, Waters) followed by an analytical reverse phase column C18 HSS T3 (100Å, 1.8µm, 75µm x 250mm; at 50°C, Waters). The peptides were eluted at a flow rate

of 300nl/min by a gradient from 5 to 35% B in 90 min, 60% B in 5 min and 80% B in 1 min. The mass spectrometer was operated in data-dependent mode (DDA), acquiring a full-scan MS spectrum (350−1'500 m/z) at a resolution of 70'000 at 200 m/z after accumulation to a target value of 3E6, followed by HCD (higher-energy collision dissociation) fragmentation on the 12 most intense signals per cycle. HCD spectra were acquired at a resolution of 35'000 using a normalized collision energy of 25 and a maximum injection time of 120ms. The automatic gain control (AGC) was set to 1E5 ions. Charge state screening was enabled. Singly and unassigned charge states were rejected. Only precursors with intensity above 2.5E4 were selected for MS/MS. Precursor masses previously selected for MS/MS measurement were excluded from further selection for 40s, and the exclusion window was set at 10ppm. The samples were acquired using internal lock mass calibration on m/z 371.1012 and 445.1200. The mass spectrometry proteomics data were handled using the local laboratory information management system (LIMS)[8].

Protein identification and label free quantification

The acquired raw MS data were processed by MaxQuant[9] (version 1.6.2.3), followed by protein identification using the integrated Andromeda search engine. Spectra were searched against a Homo Sapiens UniProt reference proteome database (taxonomy 9606; canonical version from 20190709), the reversed decoy-database and a database of common protein contaminants. Carbamidomethylation of cysteine was set as fixed, while methionine oxidation and N-terminal protein acetylation were set as variable modifications. Enzyme specificity was set to trypsin/P allowing a minimal peptide length of 7 amino acids and a maximum of two missed-cleavages. The maximum false discovery rate (FDR) was set to 0.01 for peptides and 0.05 for proteins. Label free quantification was enabled and a 0.7 min window for match between runs was applied. In the MaxQuant experimental design template, each file is kept separate in the experimental design to obtain individual intensity values.

Protein fold changes were computed based on Intensity values. A set of functions implemented in the R package SRMService (https://github.com/protViz/SRMService) was used to filter for proteins with 2 or more peptides, with reported intensities in at least 6 samples. The data were normalized with a modified robust z-score transformation to compute p-values using the t-test with pooled variance. If all measurements of a protein were missing in one of the conditions, a pseudo fold change was computed replacing the missing group average by the mean of 10% smallest protein intensities in that condition.

References to Supplementary Information

1. Kilic, S. et al. Phase separation of 53BP1 determines liquid-like behavior of DNA repair compartments. *EMBO J* **38**, e101379 (2019).
2. Frattini, C. et al. TopBP1 assembles nuclear condensates to switch on ATR signaling. *Mol Cell* **81**, 1231-1245 e8 (2021).
3. Alghoul, E., Basbous, J. & Constantinou, A. An optogenetic proximity labeling approach to probe the composition of inducible biomolecular condensates in cultured cells. *STAR Protoc* **2**, 100677 (2021).
4. Rademacher, A., Erdel, F., Trojanowski, J., Schumacher, S. & Rippe, K. Real-time observation of light-controlled transcription in living cells. *Journal of Cell Science* **130**, 4213-4224 (2017).
5. Lezaja, A. et al. RPA shields inherited DNA lesions for post-mitotic DNA synthesis. *Nat Commun* **12**, 3827 (2021).
6. Lippert, T.P. et al. Oncogenic herpesvirus KSHV triggers hallmarks of alternative lengthening of telomeres. *Nature Communications* **12**(2021).
7. Lau, L.M. et al. Detection of alternative lengthening of telomeres by telomere quantitative PCR. *Nucleic Acids Res* **41**, e34 (2013).
8. Türker, C. et al. B-Fabric: the Swiss Army Knife for life sciences. in *Proceedings of the 13th International Conference on Extending Database Technology* 717–720 (Association for Computing Machinery, Lausanne, Switzerland, 2010).
9. Cox, J. & Mann, M. MaxQuant enables high peptide identification rates, individualized p.p.b.-range mass accuracies and proteome-wide protein quantification. *Nature Biotechnology* **26**, 1367-1372 (2008).

List of Source Data Files

Numeric Source Data Figure 1
Numeric Source Data Figure 2
Numeric Source Data Figure 3
Numeric Source Data Figure 4
Numeric Source Data Figure 5
Numeric Source Data Figure 6
Numeric Source Data Extended Data Figure 1
Numeric Source Data Extended Data Figure 2
Numeric Source Data Extended Data Figure 3
Numeric Source Data Extended Data Figure 4
Numeric Source Data Extended Data Figure 5
Numeric Source Data Extended Data Figure 6
Numeric Source Data Extended Data Figure 8
Numeric Source Data Extended Data Figure 9
Numeric Source Data Extended Data Figure 10
Numeric Source Data Supplementary Figure 1
Numeric Source Data Supplementary Figure 3
Numeric Source Data Supplementary Figure 4
Uncropped Blot Source Data Figure 3
Uncropped Blot Source Data Figure 4
Uncropped Blot Source Data Extended Data Figure 2
Uncropped Blot Source Data Extended Data Figure 9
Uncropped Blot Source Data Supplementary Figure 2

Western blot scans:
Supplementary Figure 2a

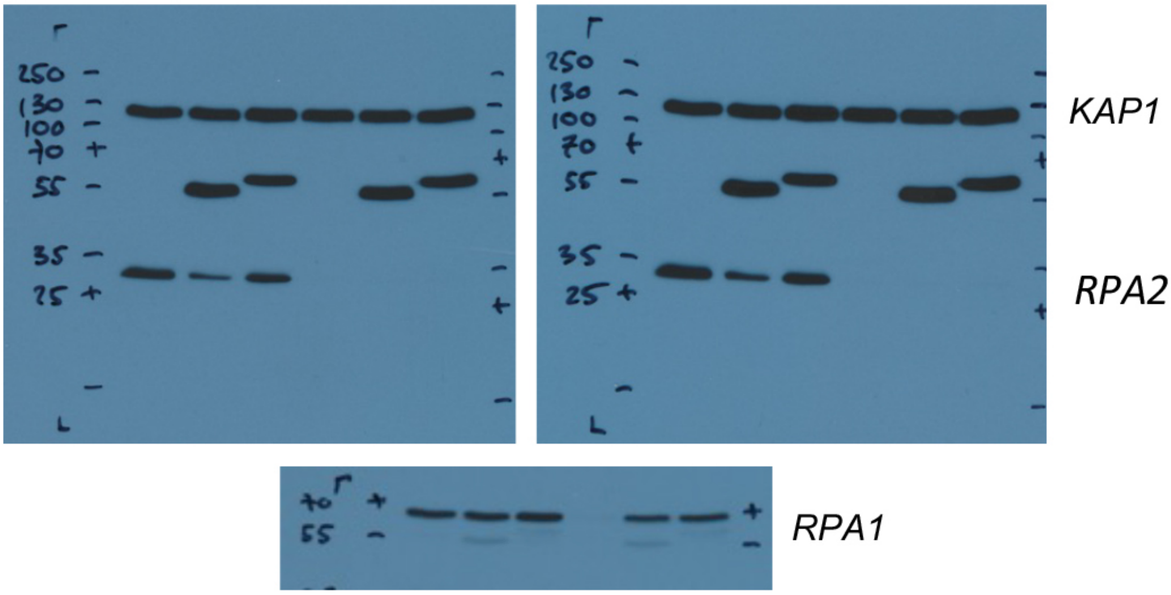

Western blot scans:
Supplementary Figure 2b

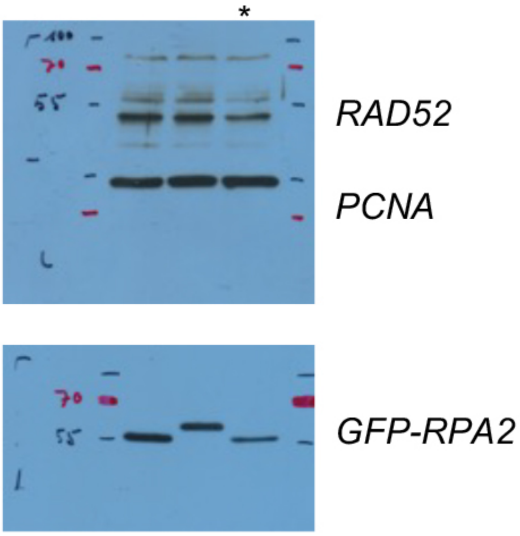

* *unrelated lane*
