# Supplementary material for: Phase separation properties of RPA combine high-affinity ssDNA binding with dynamic condensate functions at telomeres
Source: Nat Struct Mol Biol. 2023 Mar 9;30(4):451–62. doi: 10.1038/s41594-023-00932-w (PMC10113159; doi:10.1038/s41594-023-00932-w)
Supplement: Supplementary file 2 — Reporting Summary [file 41594_2023_932_MOESM2_ESM.pdf]

nature portfolio

Corresponding author(s): Matthias Altmeyer

Last updated by author(s): 01/06/2023

Reporting Summary

Nature Portfolio wishes to improve the reproducibility of the work that we publish. This form provides structure for consistency and transparency in reporting. For further information on Nature Portfolio policies, see our Editorial Policies and the Editorial Policy Checklist.

Statistics

For all statistical analyses, confirm that the following items are present in the figure legend, table legend, main text, or Methods section.

n/a	Confirmed	
☐	☒	The exact sample size (*n*) for each experimental group/condition, given as a discrete number and unit of measurement
☐	☒	A statement on whether measurements were taken from distinct samples or whether the same sample was measured repeatedly
☐	☒	The statistical test(s) used AND whether they are one- or two-sided<br>*Only common tests should be described solely by name; describe more complex techniques in the Methods section.*
☒	☐	A description of all covariates tested
☒	☐	A description of any assumptions or corrections, such as tests of normality and adjustment for multiple comparisons
☐	☒	A full description of the statistical parameters including central tendency (e.g. means) or other basic estimates (e.g. regression coefficient) AND variation (e.g. standard deviation) or associated estimates of uncertainty (e.g. confidence intervals)
☐	☒	For null hypothesis testing, the test statistic (e.g. *F*, *t*, *r*) with confidence intervals, effect sizes, degrees of freedom and *P* value noted<br>*Give P values as exact values whenever suitable.*
☒	☐	For Bayesian analysis, information on the choice of priors and Markov chain Monte Carlo settings
☒	☐	For hierarchical and complex designs, identification of the appropriate level for tests and full reporting of outcomes
☒	☐	Estimates of effect sizes (e.g. Cohen's *d*, Pearson's *r*), indicating how they were calculated

Our web collection on statistics for biologists contains articles on many of the points above.

Software and code

Policy information about availability of computer code

Data collection	High-content microscopy: ScanR Image Acquisition 3.01 and 3.2, IN Cell Analyzer 2500 V7.4<br>Metaphase spreads: Leika Application Suite X 3.7.5.24914<br>FRAP: Leika Application Suite X 3.5.7.23225<br>STED imaging: Leika Application Suite X 3.5.7.23225<br>In vitro droplet assays: Leika Application Suite X 3.5.7.23225<br>Turbidity measurements:Tecan i-control 2.0<br>Western blot film developer: Canon MP Navigator EX<br>Agarose gel imaging: Infinity ST5 Xpress<br>qPCR: Qiagen Rotor-Gene Q Series Software 20.4.21
Data analysis	Microscopy image analysis: Olympus ScanR Image Analysis Software (version 3.0.1 and version 3.2); Fiji/ImageJ 64-bit, Version 2.00-rc54/1.51h<br>Data visualization: TIBCO Spotfire data visualization software (version 7.9.1 and 10.10.1), Graphpad Prism (Version 5, 8 and 9)<br>Statistical analysis: Graphpad Prism (Version 5, 8 and 9)<br>MS: MaxQuant (Version 1.6.2.3), R package SRM Service (https://github.com/protViz/SRMService)

For manuscripts utilizing custom algorithms or software that are central to the research but not yet described in published literature, software must be made available to editors and reviewers. We strongly encourage code deposition in a community repository (e.g. GitHub). See the Nature Portfolio guidelines for submitting code & software for further information.

Data

Policy information about availability of data

All manuscripts must include a data availability statement. This statement should provide the following information, where applicable:

- Accession codes, unique identifiers, or web links for publicly available datasets
- A description of any restrictions on data availability
- For clinical datasets or third party data, please ensure that the statement adheres to our policy

The mass spectrometry proteomics data was analyzed using Homo Sapiens UniProt reference proteome database (taxonomy 9606; canonical version from 20190709), reversed decoy-database, and database of common protein contaminants. The mass spectrometry proteomics data have been deposited together with the reversed decoy-database and the database of common protein contaminants to the ProteomeXchange Consortium via the PRIDE partner repository with the dataset identifier PXD036935. Other data are provided as source data file with this paper. No restrictions apply on data availability.

Field-specific reporting

Please select the one below that is the best fit for your research. If you are not sure, read the appropriate sections before making your selection.

☒ Life sciences ☐ Behavioural & social sciences ☐ Ecological, evolutionary & environmental sciences

For a reference copy of the document with all sections, see nature.com/documents/nr-reporting-summary-flat.pdf

Life sciences study design

All studies must disclose on these points even when the disclosure is negative.

Sample size	This study did not include animal models or human participants and sample sizes were determined based on current standards in the field (e.g. Toledo et al., Cell 2013 Nov 21;155(5):1088-103; Michelena et al., Nat Commun. 2018 Jul 11;9(1):2678; Sedlackova et al., Nature, 2020 Oct 21; 587, 297-302; Lezaja et al., Nat Commun. 2021 Jun 22;12(1):3827). Exact sample sizes are provided in the methods and figure legends, and individual data points are provided in the Source Data files.
Data exclusions	No relevant data was excluded from this study.
Replication	Experiments were performed in at least 2-3 biological replicates and experimental findings were reliably reproduced. Turbo-ID proteomics was performed in six technical replicates for label-free quantification.
Randomization	Experiments were performed with asynchronously cycling cell populations and cultures serving as control or experimental groups, respectively, were randomly assigned. For each condition multiple non-overlapping fields of view using an evenly distributed standard grid were acquired.
Blinding	Data collection and analysis was conducted using automated unbiased image acquisition and analysis software. No further blinding was applied and no animals or human research participants or samples were involved in the study.

Reporting for specific materials, systems and methods

We require information from authors about some types of materials, experimental systems and methods used in many studies. Here, indicate whether each material, system or method listed is relevant to your study. If you are not sure if a list item applies to your research, read the appropriate section before selecting a response.

Materials & experimental systems

n/a	Involved in the study	
☐	☒	Antibodies
☐	☒	Eukaryotic cell lines
☒	☐	Palaeontology and archaeology
☒	☐	Animals and other organisms
☒	☐	Human research participants
☒	☐	Clinical data
☒	☐	Dual use research of concern

Methods

n/a	Involved in the study	
☒	☐	ChIP-seq
☒	☐	Flow cytometry
☒	☐	MRI-based neuroimaging

Antibodies

Antibodies used	Primary antibodies used in this study:<br>RPA70 (Abcam, ab79398, diluted 1:500 for IF and WB)<br>H2AX phospho S139 (Biolegend, 613401, diluted 1:1000 for IF)

TRF2 (Novus, NB110-57130, diluted 1:300 for IF)
RAD52 (custom-made, kindly provided by Dr. Thanos Halazonetis, diluted 1:200 for IF)
RAD52 (Santa Cruz, sc-365341, diluted 1:100 for WB)
PML (Santa Cruz, sc-966, diluted 1:1000 for IF)
RPA32 (Abcam, ab2175, diluted 1:500 for IF and WB)
RPA32 pS4/8 (Bethyl, A300-245, diluted 1:500 for WB)
CyclinA (Abcam, ab16726, diluted 1:200 for IF)
53BP1 (Novus, NB100-304, diluted 1:1000 for IF)
PCNA (Santa Cruz, sc-56, diluted 1:2000 for WB)
KAP1 (Bethyl, A300-274A, diluted 1:1000 for WB)
GFP (Torrey Pines biolabs, TP401, 0.8ug/600ug whole cell lysate for IP)

Secondary antibodies used in this study:
Alexa Fluor 647 Goat Anti-Mouse (Life Technologies, A21325, diluted 1:500 for IF)
Alexa Fluor 647 Goat Anti-Rabbit (Life Technologies, A21244, diluted 1:500 for IF)
Alexa Fluor 568 Goat Anti-Mouse (Life Technologies, A11031, diluted 1:500 for IF)
Alexa Fluor 568 Goat Anti-Rabbit (Life Technologies, A11029, diluted 1:500 for IF)
Alexa Fluor 488 Goat Anti-Rabbit (Life Technologies, A11034, diluted 1:500 for IF)
Alexa Fluor 488 Goat Anti-Mouse (Life Technologies, A11029, diluted 1:500 for IF)
Donkey anti-Sheep IgG (H+L) Cross-Adsorbed Secondary AB Alexa 647 (Life Technologies, A21448, diluted 1:500 for IF)
Goat Anti-Rabbit IgG Antibody (H+L), Peroxidase (Vector Laboratories, PI-1000-1, diluted 1:10000 for WB)
Horse Anti-Mouse IgG Antibody (H+L), Peroxidase (Vector Laboratories, PI-2000-1, diluted 1:10000 for WB)

Validation

RPA70 (Abcam, ab79398) was previously validated by immunofluorescence staining, knockdown, and by endogenous RPA tagging (Toledo et al., Cell. 21,155(5):1088-103 (2013); Lezaja et al., Nat. Commun. 12, 3827 (2021)). Furthermore it was validated by microscopic analysis of co-localization with GFP-RPA2 signal in this study. H2AX phospho S139 (Biolegend, 613401) was previously validated by ATM inhibition (Lezaja et al., Nat. Commun. 12, 3827 (2021)). TRF2 (Novus, NB110-57130) was previously validated by knockdown and telomere FISH (Smogorzewska et al., Mol. Cell Biol. 20(5):1659-68 (2000); Lezaja et al., Nat. Commun. 12, 3827 (2021)). RAD52 (custom-made) was validated by immunofluorescence staining and RAD52 knockout (Sotiriou et al., Mol Cell, 64(6): 1127-1134 (2016); Lezaja et al., Nat. Commun. 12, 3827 (2021)). RAD52 (Santa Cruz, sc-365341) was validated by western blot by the manufacturer (https://datasheets.scbt.com/sc-365431) and by knockdown (van de Kooij et al., Nat Commun. 13, 5295 (2022)). PML (Santa Cruz, sc-966) was validated previously by knockdown and used for immunofluorescence (Vilotti et al., Cell Death Differ. 2012 Mar;19(3):488-500). RPA32 (Abcam, ab2175) was previously validated by knockdown (Mylers et al., Proc Natl Acad Sci U S A. 2016 Mar;113(9):E1170-9; Lezaja et al., Nat. Commun. 12, 3827 (2021)). RPA32 pS4/8 (Bethyl, A300-245) was previously validated by western blot (Zhang et al., Nat. Commun. 13, 6907 (2022)). Cyclin A (Abcam, ab16726) was previously validated by co-staining with other cell cycle markers and used for immunofluorescence (Lyman et al., PLoS One. 7;6(3):e17692 (2011); Moreno et al., Proc Natl Acad Sci U S A. 27;113(39):E5757-64 (2016); Somyajit et al., Science. 10;358(6364):797-802 (2017); Lezaja et al., Nat. Commun. 12, 3827 (2021)). 53BP1 (Novus, NB100-304) was previously validated by the manufacturer using gamma irradiation and immunofluorescence and by knockdown (Han et al., Sci Adv. 14;1(7):e1500454 (2015)) and further validated by assessing replication stress-induced 53BP1 nuclear bodies (Lezaja et al., Nat. Commun. 12, 3827 (2021)). PCNA (Santa Cruz, sc-56) was validated by knockout and western blot (Dietsch et al. BioTechniques. 62(2):80-82 (2017)). KAP1 (Bethyl, A300-274A) was validated by the manufacturer and used in recent studies (Uhlen et al. Nat. Methods. 13(10):823 (2016); (Spies et al. Nat. Cell. Bio. 21(4):487.497 (2019); Teloni et al. Mol. Cell. 72(4):670-683 (2019); Silva et al. Nat. Commun. 10(1),2253 (2019); Ali et al. Nat. Commun. 10(1),926 (2019)) and served as loading control. GFP (Torrey Pines biolabs, TP401) was validated by western blot (Bruhn et al., Nat. Commun. 11, 4154 (2020); https://www.amsbio.com/rabbit-anti-gfp-pab-tp401). All secondary antibodies were validated by the manufacture and used in recent studies (Lezaja et al., Nat. Commun. 12, 3827 (2021), Teloni et al. Mol. Cell. 72(4):670-683 (2019), Porro et al. Sci. Adv. 7:eabf7906 (2021), Gatti et al. Cell Reports. 32,107985 (2020).

Eukaryotic cell lines

Policy information about cell lines

Cell line source(s)

U-2 OS cells: ATCC (HTB-96; RRID:CVCL_0042)
U-2 OS GFP-RPA2 WT: This study
U-2 OS GFP-RPA2 S>D: This study
U-2 OS GFP-RPA2 RFP-TRF2: This study
U-2 OS GFP-RPA: provided by Dr. Luis Toledo (University of Copenhagen, Denmark)
U-2 OS GFP-53BP1 RPA70-mScarlett: Lezaja et al., Nat Commun. 2021 Jun 22;12(1):3827 (University of Zurich, Switzerland)
U-2 OS Flp-IN Trex: provided by Dr. Kerstin Gari (Zurich University of Applied Sciences, Switzerland)
U-2 OS TurboID-RPA2-mCherry-Cry2: This study
U-2 OS TurboID-mCherry-Cry2: This study
HeLa cells: ATCC (CCL-2; RRID:CVCL_0030), kindly provided by Prof. Dr. Michael Hottiger (University of Zurich, Switzerland)

Authentication

The parental U-2 OS cell line was authenticated by STR profiling. HeLa cells used as negative control for C-circle analysis were not re-authenticated.

Mycoplasma contamination

All cell lines were tested every 4-6 weeks for mycoplasma contamination and always scored negative.

Commonly misidentified lines
(See ICLAC register)

No commonly misidentified cell lines were used in this study.
