# Supplementary figures and images for: Phase separation properties of RPA combine high-affinity ssDNA binding with dynamic condensate functions at telomeres
Source: Nat Struct Mol Biol. 2023 Mar 9;30(4):451–62. doi: 10.1038/s41594-023-00932-w (PMC10113159; doi:10.1038/s41594-023-00932-w)

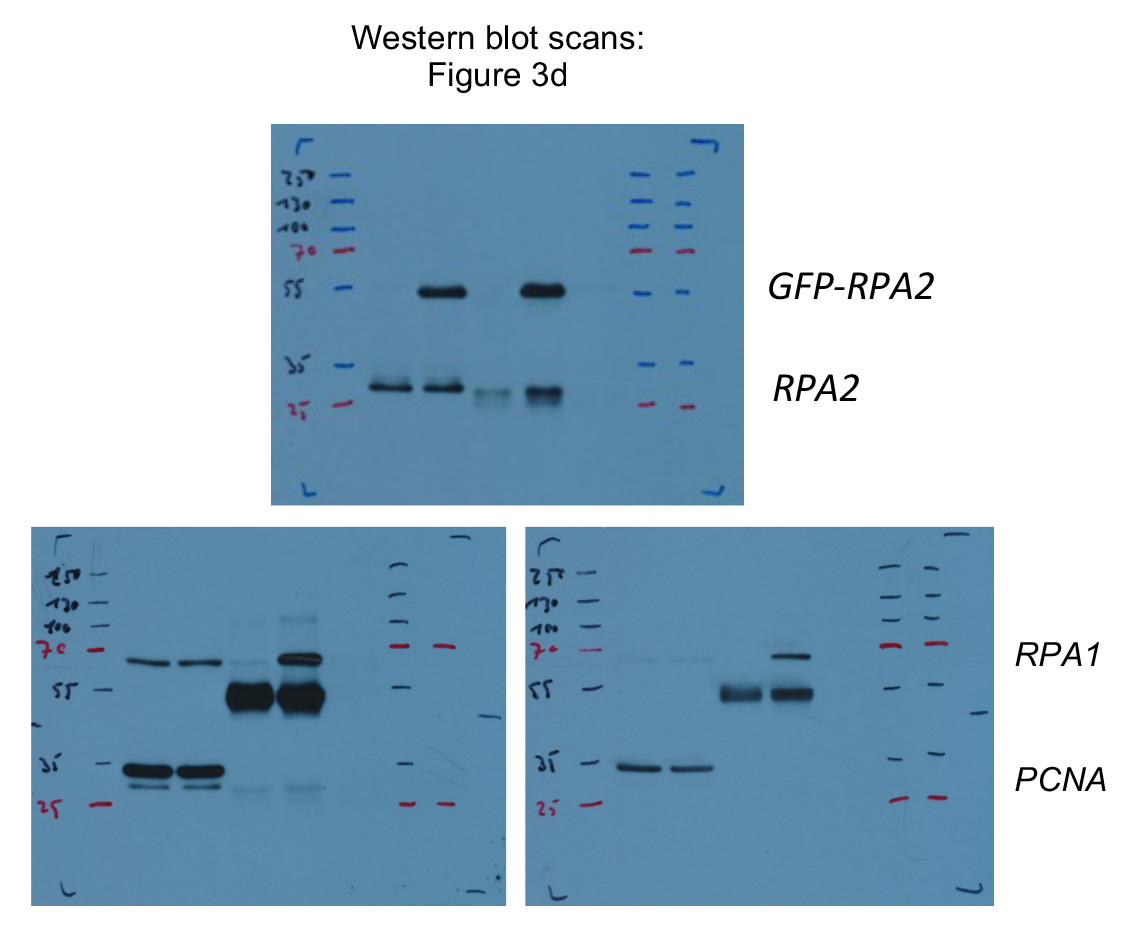

Supplement: Source Data Fig. 3 — Unprocessed Western Blots [file 41594_2023_932_MOESM11_ESM.tif]

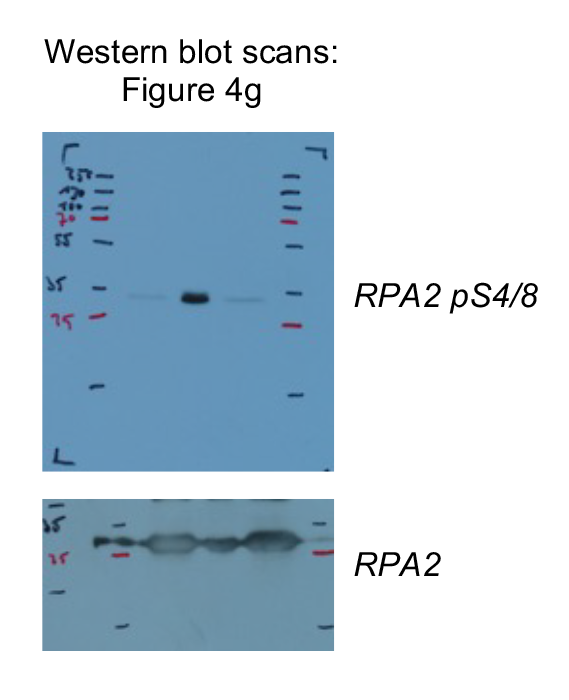

Supplement: Source Data Fig. 4 — Unprocessed Western Blots [file 41594_2023_932_MOESM13_ESM.tif]

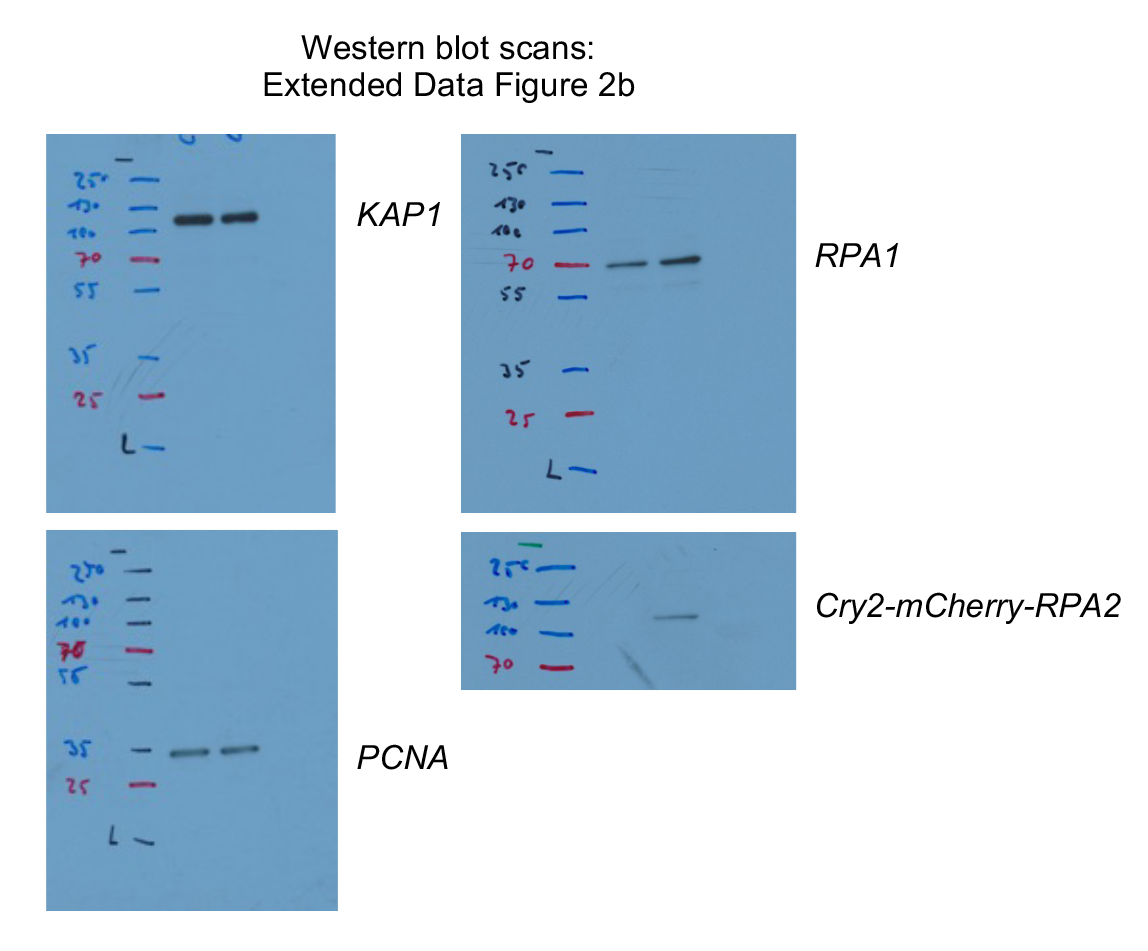

Supplement: Source Data Extended Data Fig. 2 — Unprocessed Western Blots [file 41594_2023_932_MOESM18_ESM.tif]

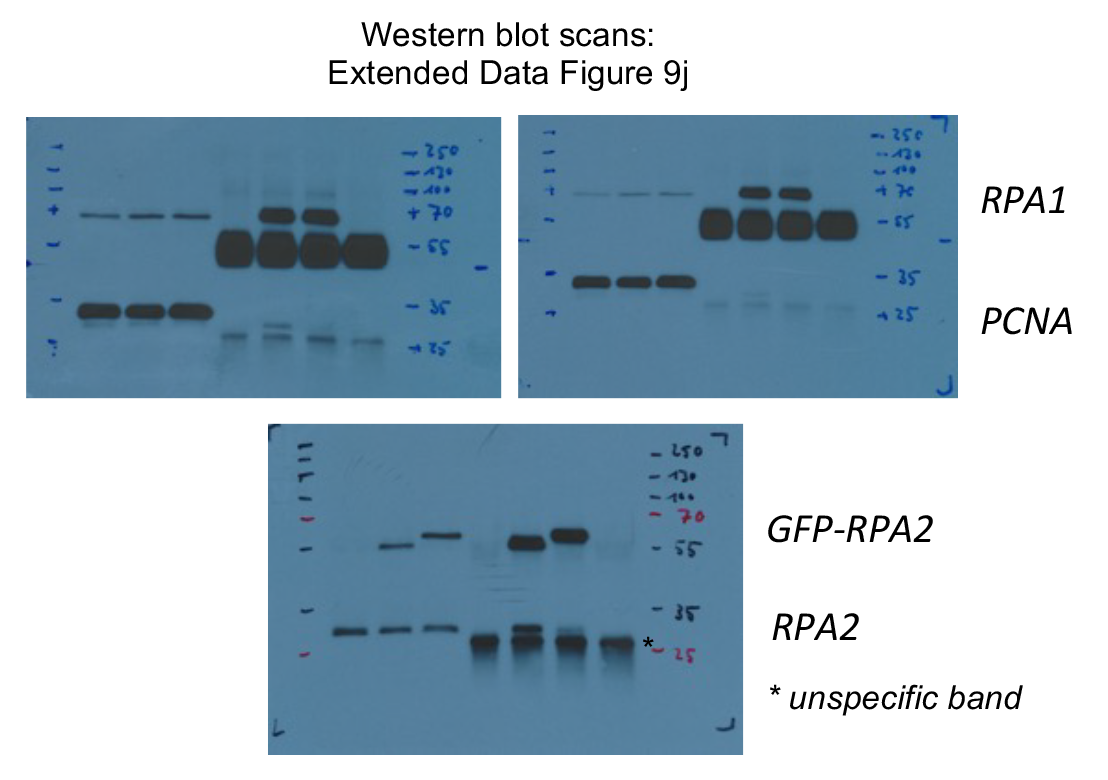

Supplement: Source Data Extended Data Fig. 9 — Unprocessed Western Blots [file 41594_2023_932_MOESM25_ESM.tif]
